# Mitochondrion-targeted therapies for diabetic wound healing: from mechanism to therapeutic opportunity

**DOI:** 10.1093/burnst/tkag018

**Published:** 2026-03-02

**Authors:** Qipeng Wu, Fei Xiao, Han Wang, Yuan Xiong, Bobin Mi

**Affiliations:** Department of Orthopedics, Union Hospital, Tongji Medical College, Huazhong University of Science and Technology, No. 1277 Jiefang Avenue, Wuhan 430022, China; Department of Orthopaedics, Wuhan Fourth Hospital, No. 473 Hanzheng Street, Wuhan 430033, China; Department of Anesthesiology, Peking University People’s Hospital, No. 7 Jinsheng First Road, Qingdao 266000, China; Department of Anesthesiology, Women and Children’s Hospital, Qingdao University, No. 217 Liaoyang West Road, Qingdao 266000, China; Department of Orthopedics, Tongji Hospital, Tongji Medical College, Huazhong University of Science and Technology, No. 1095 Jiefang Avenue, Wuhan 430030, China; Department of Orthopedics, Union Hospital, Tongji Medical College, Huazhong University of Science and Technology, No. 1277 Jiefang Avenue, Wuhan 430022, China

**Keywords:** Diabetic wounds, Mitochondrial dysfunction, Oxidative stress, Mitochondrial transfer, Therapeutic strategies

## Abstract

Diabetic wounds are a major clinical challenge. They are driven by persistent hyperglycemia and chronic inflammation that synergistically disrupt mitochondrial homeostasis, manifesting as impaired bioenergetics, excessive reactive oxygen species (ROS) accumulation, and dysregulated mitochondrial quality control. Mitochondrial dysfunction critically undermines cellular proliferation, angiogenesis, and immunomodulation, which are essential for effective tissue repair. Intercellular mitochondrial transfer, mediated through tunneling nanotubes (TNTs), extracellular vesicles (EVs), gap junctions (GJs), and cell fusion, has recently emerged as a biologically compelling endogenous rescue mechanism capable of restoring bioenergetic capacity and redox homeostasis in metabolically compromised recipient cells. In this review, we systematically examine the mechanistic basis of mitochondrial dysfunction in the diabetic wound microenvironment, critically evaluate the therapeutic potential of intercellular mitochondrial transfer, and propose an integrated mechanism-to-translational framework coupling transfer-based strategies with bioresponsive and mitochondrion-targeted biomaterials tailored to the pathological wound milieu. Furthermore, we identify key translational barriers—including insufficient protocol standardization, the absence of robust characterization criteria, and a lack of quantitative benchmarks for transfer efficacy—that must be addressed to advance these strategies toward clinical application, thereby offering a conceptual foundation and translational roadmap for mitochondrion-centered regenerative approaches in diabetic wound care.

## Highlights

- Diabetic wound chronicity arises, at least in part, from the convergent failure of mitochondrial bioenergetics, redox regulation, and quality control surveillance—a triad of dysfunction that positions mitochondrial impairment as an upstream determinant of compromised tissue repair rather than an epiphenomenal consequence of the hyperglycemic milieu.
- Tunneling nanotubes, extracellular vesicles, gap junctions, cell fusion-mediated conduits, and cell fusion collectively constitute a phylogenetically conserved intercellular mitochondrial transfer axis, the activation of which restores recipient cell bioenergetic sufficiency and immunometabolic equilibrium, thereby providing a unifying mechanistic bridge between organelle-level biology and the broader imperatives of tissue regeneration.
- Bioresponsive hydrogels, nanozyme-enabled platforms, and extracellular vesicle-integrated delivery systems have been developed to potentiate endogenous mitochondrial rescue; however, their clinical translation remains limited by the lack of validated metrics for transfer efficiency, reproducible benchmarks for delivery performance, and clearly defined criteria for assessing long-term mitochondrial integration and sustained functional activity within recipient tissues.

## Background

Diabetic wounds are a substantial clinical challenge because of their persistent inflammatory state, impaired angiogenesis, and failure to progress through the normal healing phase [1–3]. This pathology is compounded by a range of cellular and molecular dysfunctions, notably mitochondrial dysfunction, which has emerged as a critical factor in the impaired healing process of diabetic wounds [1, 4, 5]. Mitochondria, often referred to as the powerhouses of cells, are integral to cellular energy metabolism, apoptosis regulation, and oxidative stress responses [4, 6]. In the context of diabetes, mitochondrial dysfunction manifests as impaired wound healing, heightened oxidative stress, and diminished cellular regeneration, positioning mitochondrial function as a pivotal point for therapeutic intervention [5, 7].

Recent studies emphasize the potential of mitochondrial transfer between cells as a mechanism to restore cellular function and promote healing in diabetic wounds [8]. Mitochondrial transfer can occur through several mechanisms, including tunneling nanotubes (TNTs), extracellular vesicles (EVs), gap junctions (GJs), and cell fusion [9–12]. This intercellular exchange not only augments the energy production and stress response capabilities of recipient cells but also enhances critical cellular processes for wound healing, such as angiogenesis and macrophage polarization [13, 14]. While these studies collectively support mitochondrial transfer as a reparative mechanism in diabetic wounds, emerging evidence also reveals substantial heterogeneity in transfer efficiency, dominant pathways, and functional durability across experimental systems, underscoring the need for a more integrated and critical evaluation of this phenomenon.

Innovative materials and strategies are being investigated to facilitate mitochondrial transfer and thereby improve diabetic wound healing [5, 15, 16]. Among these are biohydrogels, mesenchymal stem cell (MSC)-derived EVs, and self-healing conductive hydrogels. Additionally, therapies targeting mitochondrial dynamics—such as inhibiting mitochondrial fission or modulating mitochondrial autophagy—have shown promise in restoring mitochondrial function and accelerating tissue repair [17, 18]. Collectively, these findings suggest that mitochondrial dysfunction represents an upstream contributor to impaired healing, which in turn frames mitochondrial transfer or transplantation as a potential downstream approach to restore cellular metabolic competence.

The role of mitochondrial transfer in diabetic wound healing is complex and multifaceted. For instance, MSC-derived EVs, which contain functional mitochondria, have been shown to expedite wound healing by enhancing endothelial cell function and promoting the polarization of macrophages toward the pro-healing M2 phenotype [19, 20]. Furthermore, novel technology approaches employing acid-responsive nanoparticles and dual-responsive hydrogels are being developed to target and repair mitochondrial dysfunction in endothelial cells and fibroblasts, thus optimizing the wound healing process [5, 21].

This review synthesizes current evidence on mitochondrial dysfunction and intercellular mitochondrial transfer in diabetic wound healing and frames mitochondrion-targeted interventions within an integrated mechanism-to-translational paradigm that connects mitochondrial quality control, endogenous and engineered transfer pathways, and bioresponsive biomaterials while delineating critical translational challenges for the development of future therapeutic opportunities in chronic diabetic wounds.

## Review

### Mitochondrial dysfunction in diabetic wound healing

**Figure 1:**
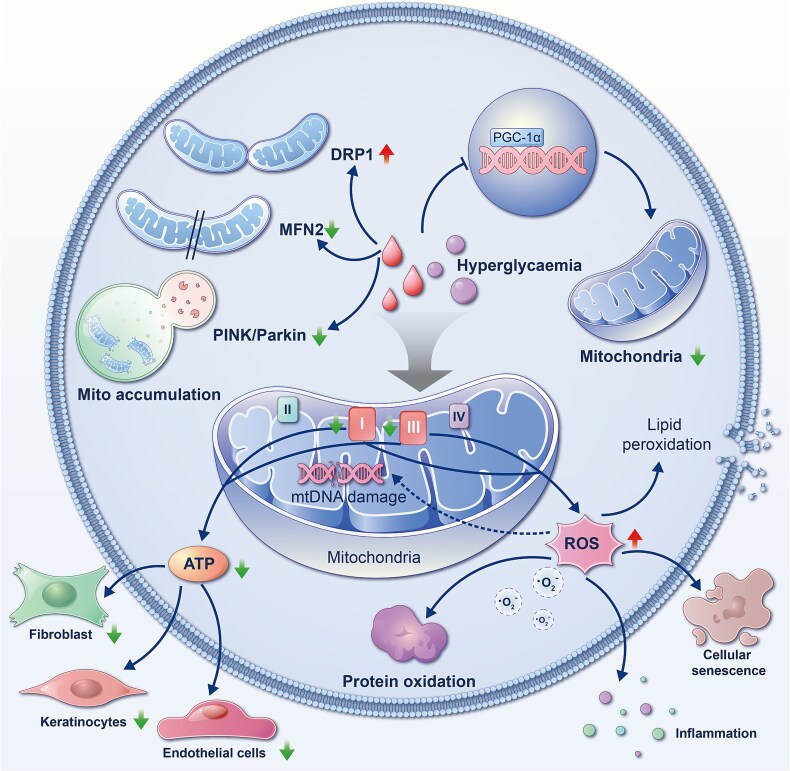
Schematic illustration of mitochondrial dysfunction in diabetic wound healing. A magnified mitochondrion within a semitransparent cell highlights complexes I–IV, with complexes I and III impaired. Hyperglycemia negatively affects these complexes, lowering ATP levels and impairing fibroblasts, keratinocytes, and endothelial cells. Electron leakage from complexes I and III generates ROS, causing lipid, protein, and mtDNA damage while triggering inflammation and senescence. Mitochondrial quality control is disrupted by the suppression of PGC-1α, increased expression of Drp1, decreased expression of MFN2, and reduced expression of PINK1/Parkin, leading to the accumulation of damaged mitochondria. This integrated diagram visually summarizes the multifactorial mitochondrial impairments contributing to delayed diabetic wound healing. *ATP* adenosine triphosphate, *mtDNA* mt deoxyribonucleic acid, *PGC-1α* peroxisome proliferator-activated receptor gamma coactivator 1-alpha, *Drp1* dynamin-related protein 1, *MFN2* mitofusin 2, *PINK1* PTEN-induced kinase 1. Created with Adobe illustrator

Mitochondria are central to cellular energy production, redox regulation, and signaling [22–24], all of which are indispensable for effective wound repair. In diabetic wounds, persistent hyperglycemia and chronic inflammation disrupt mitochondrial homeostasis, leading to structural and functional alterations that contribute to delayed healing [25, 26]. The pathological changes include impaired energy metabolism, excessive oxidative stress, and dysregulation of mitochondrial quality control (Figure 1).

#### Mitochondrial abnormalities in diabetic wound healing

Impaired mitochondrial metabolism constitutes a core pathological feature of diabetic wounds [27, 28]. Under physiological conditions, oxidative phosphorylation (OXPHOS) sustains ATP production to support cell proliferation, migration, and extracellular matrix (ECM) synthesis. In the diabetic wound environment, however, dysfunction of mitochondrial respiratory chain complexes—most notably complexes I and III—reduces OXPHOS efficiency and ATP availability, accompanied by a metabolic shift toward less efficient glycolysis [29]. This energetic insufficiency undermines fibroblast contractility, keratinocyte re-epithelialization, and endothelial angiogenic responses, thereby delaying wound closure [30, 31].

In parallel with energy failure, mitochondrial redox imbalance critically amplifies cellular injury in diabetic wounds [32, 33]. Chronic hyperglycemia enhances electron leakage from the respiratory chain, driving the excessive production of mitochondrial reactive oxygen species (ROS), particularly at complexes I and III [34, 35]. Pathologic ROS accumulation induces lipid and protein oxidation as well as mitochondrial deoxyribonucleic acid (mtDNA) damage, further destabilizing respiratory chain integrity and reinforcing a self-perpetuating cycle of oxidative stress and bioenergetic decline [36, 37]. Beyond direct mitochondrial injury, sustained redox dysregulation activates inflammatory signaling pathways and promotes stress-induced cellular senescence [38], preventing the orderly transition from inflammation to proliferation during wound repair [39–41].

These metabolic and oxidative insults are further exacerbated by disruption of mitochondrial quality control systems [42–44]. In diabetic wounds, suppression of PGC-1α–dependent mitochondrial biogenesis limits organelle renewal, whereas an imbalance between excessive fission and insufficient fusion leads to mitochondrial fragmentation and loss of network integrity [45–48]. Concurrent impairment of PINK1/Parkin-mediated mitophagy permits the accumulation of dysfunctional mitochondria, amplifying ROS production and energy production failure [49, 50]. Recent evidence further implicates mtDNA-centered stress responses in this process, as oxidative injury and defective mitophagy facilitate mtDNA release and the activation of innate immune pathways such as cGAS–STING [51–53]. In addition, mitochondrial-derived peptides, including MOTS-c and humanin, have emerged as regulators of metabolic stress and inflammatory responses [54, 55]. Collectively, these interconnected abnormalities disrupt mitochondrial homeostasis across keratinocytes, fibroblasts, endothelial cells, and macrophages, reinforcing chronic inflammation, cellular senescence, and impaired progression of the wound-healing cascade [14, 56–58].

#### Impact of mitochondrial dysfunction on cellular function

Mitochondrial dysfunction has been increasingly implicated in impaired diabetic wound healing and is associated with alterations in multiple interconnected cellular processes across key wound-relevant cell types, including keratinocytes, fibroblasts, endothelial cells, and macrophages [59]. Disruption of these processes is thought to contribute to defective progression through the wound repair cascade [60].

In multiple cell types (keratinocytes, fibroblasts, endothelial cells, and macrophages), mitochondrial dysfunction in diabetic wounds is associated with a limited set of recurring abnormalities, most notably bioenergetic insufficiency, excessive mtROS accumulation, and impaired mitochondrial quality control. The major mitochondrial alterations and their functional consequences in each cell type are summarized in Table 1.

**Table 1:** Mitochondrial dysfunction-associated alterations and functional consequences across cell types in diabetic wounds

Cell type	Dominant mitochondrial abnormalities	Functional consequence	Implication for diabetic wounds healing	References
Keratinocytes	ATP deficiency; excessive ROS production; impaired mitophagy and mitochondrial biogenesis	Reduced proliferation and migration; increased apoptosis/senescence	Delayed re-epithelialization	[64–66]
Fibroblasts	Bioenergetic insufficiency; ROS-driven damage; mitochondrial fragmentation	Decreased collagen synthesis and contractility; cellular senescence	Impaired granulation tissue formation and ECM remodeling	[67–69]
Endothelial cells	Reduced OXPHOS capacity; mtROS-mediated nitric oxide dysregulation; mitochondrial quality control failure	Defective tube formation and survival; pro-inflammatory phenotypic shift	Impaired angiogenesis and tissue perfusion	[70–72]
Macrophages	Respiratory dysfunction; defective mitophagy; mtDNA-linked inflammatory amplification	Sustained M1 polarization; inflammasome activation; excessive cytokine production	Chronic inflammation and failure to transition to repair	[52, 63, 73]

*ATP* adenosine triphosphate, *ROS* reactive oxygen species, *ECM* extracellular matrix, *OXPHOS* oxidative phosphorylation

Mitochondrial dysfunction exerts cell type-specific yet convergent inhibitory effects across multiple wound-relevant cell populations, collectively undermining coordinated tissue repair in diabetic wounds [61–63]. In keratinocytes, disrupted mitochondrial homeostasis interferes with cytoskeletal remodeling and survival- and cell cycle-related signaling pathways, resulting in reduced migratory capacity and delayed re-epithelialization, thereby directly compromising epidermal regeneration [64–66]. In fibroblasts, mitochondrial abnormalities are functionally associated with impaired ECM synthesis, defective myofibroblast differentiation, and insufficient wound contraction, which together delay the formation of granulation tissue and hinder the matrix remodeling required for timely wound closure [67–69]. Endothelial cells are likewise highly sensitive to mitochondrial impairment, as compromised mitochondrial function limits endothelial proliferation, migration, and tube formation, ultimately restricting angiogenic competence and impairing oxygen and nutrient delivery to the regenerating tissue [70–72]. In parallel, mitochondrial dysfunction in macrophages disrupts immunometabolic programming, promoting sustained proinflammatory activation while limiting the transition toward reparative, M2-like phenotypes that are essential for inflammation resolution, angiogenesis, and tissue remodeling [52, 63, 73]. Collectively, these mitochondrial defects across epidermal, mesenchymal, vascular, and immune compartments converge to stabilize a nonhealing wound microenvironment, highlighting mitochondrial dysfunction as a unifying pathological driver of impaired diabetic wound healing [61, 63, 74].

Taken together, these findings reveal that mitochondrial dysfunction emerges not merely as a downstream consequence of diabetic pathology but also as an upstream driver of impaired healing, providing a mechanistic rationale for mitochondrial transfer and transplantation as targeted restorative strategies rather than adjunctive interventions.

### Mechanisms of mitochondrial transfer

Mitochondrial dysfunction in diabetic wounds arises from impaired mitochondrial dynamics and quality control, exacerbating oxidative stress, immune dysregulation, and defective repair. Recent evidence indicates that this dysfunction extends to pathological intercellular export of mitochondrial damage via hyperglycemia-induced mitochondrial-derived vesicles, which propagate metabolic stress and delay healing [5]. In this context, intercellular mitochondrial transfer is increasingly viewed as a compensatory mechanism to restore mitochondrial integrity and cellular function.

In addition to restoring cellular bioenergetics and attenuating oxidative stress, mitochondrial transfer has been linked to the activation of downstream signaling pathways that reprogram recipient cell function. Enhanced mitochondrial membrane potential and redox homeostasis are associated with the engagement of energy-sensing and transcriptional programs, including AMPK–PGC-1α signaling, in addition to the suppression of mtROS-driven inflammatory pathways such as NF-κB and NLRP3 [75–77]. In a cell type-dependent manner, these signaling events further intersect with angiogenic and immunometabolic axes, including HIF-1α–VEGF signaling in endothelial cells [78] and metabolic polarization pathways in macrophages, thereby coordinating tissue repair responses [76, 79].

#### Overview of mitochondrial dynamics and their role in cellular function

**Figure 2:**
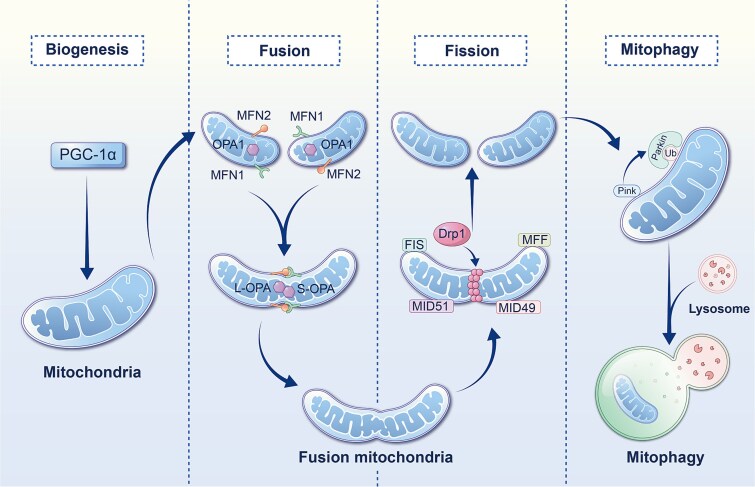
Overview of mitochondrial quality control. Mitochondrial function is preserved through an integrated network of regulatory processes, including biogenesis, fusion, fission, and mitophagy. Biogenesis, largely driven by PGC-1α, promotes the generation of new mitochondria to meet cellular energy demands. Fusion, mediated by MFN1, MFN2, and OPA1, facilitates the exchange of mitochondrial contents, supporting functional complementation and adaptation to stress. Fission, coordinated by Drp1 and its adaptors (FIS1, MFF, MID49, and MID51), enables mitochondrial distribution, segregation of damaged organelles, and cellular remodeling. Mitophagy, which is governed primarily by the PINK1–Parkin pathway, selectively removes dysfunctional mitochondria through lysosomal degradation. Together, these processes maintain mitochondrial integrity, bioenergetic capacity, and cellular homeostasis, which are critically involved in tissue repair and pathological conditions. *MFN* mitofusin, *OPA1* optic atrophy 1, *FIS1* fission, mitochondrial 1, *MFF* mitochondrial fission factor, *MID* mitochondrial dynamics protein. Created with Adobe illustrator

Mitochondrial dynamics, referring to the continuous changes in mitochondrial morphology, number, and intracellular localization, encompass key processes such as fission, fusion, biogenesis, and mitophagy (Figure 2). These processes are essential for maintaining cellular homeostasis and function, including ATP production and the regulation of fundamental biological activities such as cell motility, differentiation, cell cycle progression, senescence, and apoptosis [80, 81].

Mitochondrial quality control functions as an integrated regulatory network in which fission facilitates the segregation of damaged mitochondrial components for PINK1–Parkin–dependent clearance [82, 83], whereas fusion and biogenesis, coordinated by the PGC-1α–TFAM axis, replenish respiratory capacity and preserve mitochondrial genome integrity [84]. In diabetic wounds, the concurrent suppression of mitochondrial biogenesis, dysregulation of Drp1–MFN2-mediated dynamics, and impaired mitophagy converge to promote persistent mtROS accumulation, mtDNA damage, and inflammatory signaling, thereby reinforcing the chronic, nonresolving wound microenvironment [85, 86].

In addition to their structural and energetic roles, mitochondrial dynamics are closely linked to the regulation of cellular metabolism, particularly in immune cells [87, 88]. For example, macrophages polarized to a proinflammatory M1 phenotype rely predominantly on glycolysis, whereas alternatively activated M2 macrophages—which are crucial for tissue repair and wound healing—primarily utilize enhanced OXPHOS [89]. These dynamic changes enable cells to adapt to various physiological and pathological stressors and are intricately involved in the regulation of energy metabolism, apoptosis, and ROS generation [90]. Overall, mitochondrial function is fundamental to a wide range of cellular processes, including metabolic regulation, intracellular signaling, and cell survival.

##### Mitochondrial fission and fusion

Mitochondrial dynamics, encompassing the processes of fission and fusion, are essential for regulating mitochondrial morphology, quantity, and distribution, thereby ensuring efficient energy production and cellular metabolic homeostasis [91, 92]. Fusion supports OXPHOS, enhances ATP synthesis, and protects mitochondria from degradation, whereas fission facilitates mitochondrial quality control and redistribution during cell division [93, 94]. Dysregulation of these processes—often due to mutations in key regulatory genes such as MFN1, MFN2, or DNM1L—leads to impaired mitochondrial bioenergetics, mtDNA depletion, increased ROS, and suppressed ATP generation [92, 95]. Such imbalances contribute to metabolic dysfunction and are implicated in a spectrum of mitochondrial diseases, collectively termed disorders of mitochondrial dynamics [48]. These include neuromuscular syndromes, metabolic disorders, and diabetes [96]. Collectively, these findings emphasize the critical role of mitochondrial dynamics in maintaining cellular energy balance and systemic metabolic regulation.

##### Mitochondrial biogenesis

Mitochondrial homeostasis is maintained through a dynamic balance between mitochondrial biogenesis and mitophagy, both of which are essential for adapting to cellular energy demands and environmental stress [97, 98]. Mitochondrial biogenesis, which is regulated by key molecules such as PGC-1α, involves the synthesis of new mitochondria to increase oxidative capacity, particularly in metabolically active tissues such as skeletal muscle, neurons, and wounded tissue [99, 100]. This process ensures adequate ATP production and redox balance during cellular proliferation, regeneration, and repair. In contrast, mitophagy selectively removes damaged or dysfunctional mitochondria, preventing excessive ROS accumulation and mitochondrion-mediated apoptosis [83]. Dysregulation of either pathway can disrupt mitochondrial quality control, contributing to impaired tissue regeneration, as seen in chronic wounds and age-related neuromuscular decline. Thus, therapeutic strategies that enhance mitochondrial biogenesis or promote mitophagy hold promise for improving mitochondrial function and tissue repair in pathological contexts requiring large amounts of energy.

Mitochondrial dynamics, biogenesis, and mitophagy operate as an integrated quality control network that preserves mitochondrial integrity under physiological conditions [84]. In the diabetic wound microenvironment, the suppression of PGC-1α–TFAM–dependent biogenesis [101], an imbalance in DRP1–MFN2–mediated dynamics [102], and impaired PINK1–Parkin–dependent mitophagy become uncoupled and mutually reinforcing [103]. This coordinated failure promotes persistent bioenergetic insufficiency, excessive mtROS accumulation and sustained inflammatory signaling, thereby stabilizing the chronic nonhealing wound state [104].

#### Major modes of mitochondrial transfer

**Figure 3:**
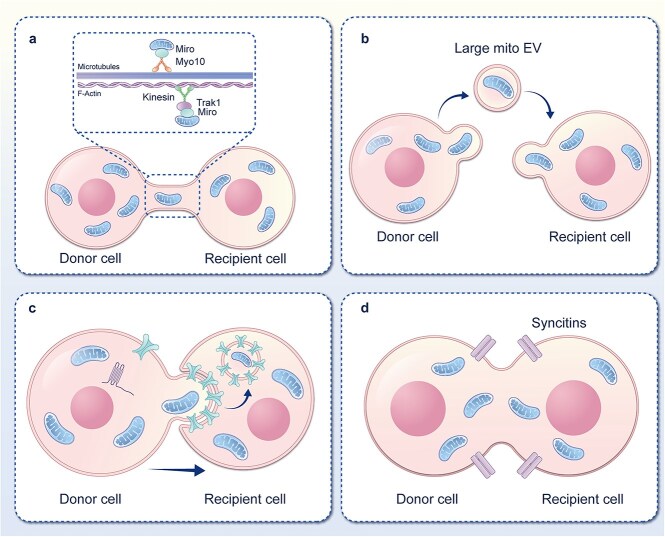
Modes of intercellular mitochondrial transfer. (**a**) TNTs establish direct cytoplasmic continuity between donor and recipient cells via actin filaments and microtubules, enabling active mitochondrial trafficking driven by molecular motors including myosin, kinesin, and dynein, in coordination with adaptor proteins of the Miro/TRAK (Trak1/Trak2) complex. (**b**) EVs mediate long-range mitochondrial exchange between spatially separated cells. Mitochondria encapsulating EVs are generated by budding or secretion from donor cells and subsequently internalized by recipient cells through plasma membrane fusion, enabling mitochondrial delivery across substantial intercellular distances without direct cell contact. (**c**) GJs facilitate organelle transfer at sites of direct cell–cell contact. Cx43 assembled GJs at cell–cell contact sites form intercellular channels that permit the passage of mitochondria or mitochondria-derived components between adjacent cells. (**d**) Cell fusion represents an additional mechanism for mitochondrial sharing. Mitochondrial sharing occurs during transient or permanent plasma membrane fusion between two cells, a process dependent on the surface expression of fusion protein, most notably syncytins which are required to drive complete membrane merger and cytoplasmic exchange. *Miro* mitochondrial rho, *Trak* trafficking kinesin protein, *Cx43* connexin 43, *EVs* extracellular vesicles. Created with Adobe illustrator

Mitochondrial transfer between cells is closely intertwined with mitochondrial dynamics, including fission, fusion, biogenesis, and mitophagy [4, 105, 106]. These dynamic processes maintain mitochondrial quality, size, and morphology, which are essential for determining whether mitochondria are suitable for intercellular transport and functional integration. Proper mitochondrial dynamics not only ensure cellular homeostasis but also facilitate the selective packaging and mobilization of healthy mitochondria for transfer. Intercellular mitochondrial transfer plays pivotal roles in tissue repair, immune regulation, and cellular rescue, particularly under pathological conditions such as injury, inflammation, or metabolic stress. It serves as a compensatory mechanism through which damaged or energy-deficient cells receive functional mitochondria from neighboring or stem cells to restore metabolic competence. Intercellular mitochondrial transfer is increasingly recognized as a compensatory mechanism in pathological contexts, including injury, inflammation, and metabolic stress, through which damaged or energy-deficient cells acquire functional mitochondria from neighboring or stem cells to support tissue repair, immune regulation, and metabolic recovery. This process occurs through four major pathways—TNTs, EVs, GJs, and cell fusion—each involving distinct forms of direct or indirect cell–cell communication that will be further discussed below (Figure 3). In addition to their structural diversity, mitochondrial transfer mechanisms have been linked to functional recovery in diabetic wounds. Across wound-relevant cell types and experimental models, mitochondrial transfer is associated with improved mitochondrial bioenergetics, reduced inflammatory stress, and enhanced angiogenesis and wound closure.

##### Tunneling nanotubes

TNTs, first identified by Rustom *et al.* in 2004 [12], are long, membranous structures that establish direct connections between cells over considerable distances, enabling the intercellular transfer of various organelles, including mitochondria. This unique cellular communication system plays a pivotal role in cellular rescue and metabolic support under pathophysiological conditions. In diabetic wounds, which are typically characterized by chronic inflammation, elevated levels of ROS, persistent hyperglycemia, hypoxia, and impaired angiogenesis, mitochondrial dysfunction represents a significant barrier to effective tissue regeneration [107]. The transfer of mitochondria via TNTs has been demonstrated to restore mitochondrial function in recipient cells, improving their bioenergetic status and reducing oxidative stress, which collectively enhances cell survival and promotes wound healing. Furthermore, mitochondrial transfer through EVs, such as exosomes, provides an alternative route for mitigating the damaging effects of the diabetic wound microenvironment. This mechanism further supports cellular metabolism, helping to counteract the detrimental conditions present in chronic wounds. These findings highlight the therapeutic potential of TNT-mediated mitochondrial transfer as a promising strategy for improving wound healing in diabetic patients and offer novel insights into potential interventions aimed at restoring mitochondrial function in compromised tissues.

##### Extracellular vesicles

Cells secrete small, membrane-bound particles known as EVs, which are essential mediators of intercellular communication [108, 109]. EVs are a heterogeneous population of lipid bilayer-enclosed nanoparticles that vary in origin, size, and morphology. On the basis of their biogenesis and physical characteristics, EVs can be classified into distinct subtypes, including exomeres (<50 nm), exosomes (30–150 nm), microvesicles (100–1000 nm), ectosomes (100–1000 nm), platelet-derived microparticles (PMPs, 100–1000 nm), apoptotic bodies (1000–5000 nm), migrasomes (500–3000 nm), and large oncosomes (1000–10 000 nm) [10, 108, 110, 111]. Under stress or injury, cells can selectively package mitochondrial components into EVs—referred to as mitochondrial-derived EVs (mitoEVs)—in a tightly regulated manner [112]. These mitoEVs may carry a diverse array of mitochondrial cargo, including mtDNA fragments or full-length mtDNA, mitochondrial proteins, and even intact mitochondria, depending on the vesicle subtype [113–116]. MitoEVs contribute to cellular adaptation to oxidative stress, inflammation, and injury. Given that mitochondrial dysfunction is associated with oxidative stress, insulin resistance, and metabolic disorders [117], the restoration of mitochondrial function has emerged as a promising therapeutic strategy to enhance tissue repair in diabetic wounds [118]. Notably, EVs facilitate the transfer of functional mitochondria to recipient cells, thereby improving their metabolic activity and promoting vascular cell proliferation, a critical process for diabetic wound healing [119, 120].

##### Gap junctions

GJs are specialized clusters of intercellular channels composed of connexin (Cx) family transmembrane proteins, among which connexin 43 (Cx43) is particularly prominent in mediating mitochondrial transfer [121–124]. In addition to its canonical role in electrical and metabolic coupling, Cx43 participates in various physiological processes, including vesicle trafficking, mitochondrial respiration, and cellular stress responses. Recent studies utilizing three-dimensional electron microscopy and immunogold labeling techniques have directly visualized Cx43-mediated mitochondrial transfer *in vivo* [125]. This horizontal mitochondrial transfer has been observed via Cx43-containing GJs in several physiological contexts, such as within ovarian follicles [126] and from hematopoietic progenitor cells to bone marrow stromal cells [124]. Moreover, the internalization of GJs can facilitate the intercellular exchange of larger cytoplasmic components, including mitochondria, endosomes, and ATP molecules [125]. GJs have been proposed to contribute to intercellular mitochondrial rescue; however, their role in direct mitochondrial transfer remains debated. Accumulating evidence indicates that GJs primarily mediate the exchange of ions and small metabolites through gap junction intercellular communication rather than through the direct passage of intact mitochondria [127]. Any involvement of GJs in mitochondrial transfer is therefore more likely to occur indirectly, for example, through connexin-mediated metabolic coupling or gap junction internalization processes that may facilitate subsequent mitochondrial exchange or functional rescue [123]. Although increasing evidence supports a role for Cx43-containing GJs in intercellular mitochondrial transfer, the underlying mechanism remains incompletely defined.

##### Cell fusion

Cell fusion is a biological process through which two individual cells merge their plasma membranes and cytoplasmic contents, ultimately forming a single, multinucleated hybrid cell [128]. This fusion can occur spontaneously under physiological or pathological conditions or can be artificially induced. Upon membrane fusion, organelles, including mitochondria, may be transferred either entirely or partially between cells, facilitating mitochondrial exchange [129]. Cell fusion can be categorized as homotypic—between identical cell types—or heterotypic—between distinct cell types. While homotypic fusion is critical for normal physiological processes such as placentation and skeletal muscle development, both homotypic and heterotypic fusion events have been reported in tumors and within the tumor microenvironment, involving cancer cells and MSCs [130–132], macrophages [133, 134], fibroblasts [135–137], and endothelial cells [138]. Syncytins—endogenous retroviral envelope proteins—are the only known fusogens expressed in humans and are essential molecular mediators of cell fusion. Notably, their upregulation in tumors such as neuroblastoma and endometrial carcinoma highlights their potential as biomarkers of cell fusion activity [9]. Additionally, cytoskeletal remodeling via the GTPases Rac1 and Cdc42 and the Arp2/3–WASP complex orchestrates the actin-driven membrane dynamics and motility essential for fusion. Importantly, fusion events significantly increase mitochondrial delivery, thereby enhancing cellular bioenergetics. Although rare under physiological conditions, cell fusion frequency increases markedly in response to stressors such as hypoxia-induced apoptosis [139], chronic inflammation [140], and ionizing radiation [141]. Increased fusion between MSCs and damaged somatic cells promotes mitochondrial transfer, contributing to heterokaryon formation and tissue regeneration. Although cell fusion is not a ubiquitous process across all tissues under physiological conditions, it is an essential and highly active mechanism in specific developmental and homeostatic contexts, such as skeletal muscle formation and placental syncytiotrophoblast development. In addition to these specialized contexts, cell fusion generally occurs at relatively low frequencies and is often associated with tissue injury, regeneration, or pathological remodeling.

Importantly, the mitochondrial transfer mechanisms described above provide a mechanistic framework for the design of mitochondrion-targeted therapeutic strategies. Many emerging interventions discussed in the following section—including biomaterial-assisted mitochondrial delivery, metabolic modulation, and bioresponsive platforms—can be viewed as attempts to harness or emulate endogenous mitochondrial transfer pathways to restore mitochondrial homeostasis in diabetic wounds. This mechanistic–therapeutic continuum enables a more integrated understanding of how insights into mitochondrial dynamics and intercellular exchange can be translated into rational therapeutic approaches.

### Biotechnology for mitochondrial rescue and transfer

Building on insights into endogenous mitochondrial dysfunction and intercellular mitochondrial transfer, the therapeutic strategies discussed in this section are organized according to their primary biological mechanisms of action rather than material composition [142]. Despite substantial diversity in platform design, most mitochondrion-targeted interventions for diabetic wound healing converge on a limited set of core objectives, including scavenging pathological mitochondrial ROS [143], restoring mitochondrial dynamics [86], enhancing mitochondrial biogenesis [101], and enhancing mitochondrial quality control [103]. Framing these approaches through their shared mechanistic basis highlights their conceptual innovation and facilitates comparison across different technological implementations.

Mitochondrion-targeted interventions have increasingly focused on mitochondrial transfer and transplantation to restore mitochondrial function and reprogram dysfunctional wound microenvironments. In recent years, various mitochondrion-targeted technologies have been developed, including both direct mitochondrial transplantation and indirect transplantation via biomaterials and nanoplatforms (Table 2). Accordingly, the biotechnological strategies described below should not be viewed solely as indirect mitochondrial regulators but rather as complementary approaches that either facilitate intercellular mitochondrial transfer, enhance the functional integration and persistence of transferred mitochondria, or recapitulate key downstream effects of mitochondrial transfer, including suppression of oxidative stress and restoration of metabolic homeostasis.

**Table 2:** Comparative analysis of mitochondrial transplantation technologies

Biotechnology type	Mechanism	Advantages	Challenges or limitations	Reference
Direct mitochondrial transplantation	Delivery of functional mitochondria directly into damaged tissues to restore cellular energy metabolism	Rapid restoration of mitochondrial function; well-defined biological effects	Immunogenicity concerns; mitochondrial degradation; low delivery efficiency	[145, 147]
Hydrogel-based delivery platforms	Encapsulation of bioactive molecules within smart hydrogels to modulate mitochondrial activity	Localized delivery; controlled release; multifunctional integration	Limited biostability; potential material toxicity; complex preparation procedures	[86, 143, 148]
Nanoparticle delivery systems	Targeted delivery to mitochondria to alleviate oxidative stress and improve mitochondrial function	High targeting specificity; regulation of ROS and mitochondrial membrane potential	Potential toxicity; biodegradability concerns; delivery efficiency optimization needed	[5, 146]
Extracellular vesicle-mediated transfer	Use of MSC-derived EVs to deliver mitochondria or mitochondrial components, modulating immunity and restoring function	Low immunogenicity; natural delivery capability	Limited cargo capacity; difficulties in isolation and purification	[19, 151]
Small molecule/natural compound modulation	Activation of pathways such as AMPK or STAT6 to reprogram immune metabolism and improve mitochondrial function	High safety profile; suitable for chronic inflammatory conditions	Limited duration of efficacy; mechanisms require further elucidation	[152]
Phototherapy-enhancement of mitochondrial function	Use of LED light to activate mitochondrial metabolism, promoting angiogenesis and tissue repair	Noninvasive; potentially applicable in home-based therapy	Limited tissue penetration; restricted clinical indications	[74]

*AMPK* activated protein kinase, *STAT6* signal transducer and activator of transcription 6, *EVs* extracellular vesicles

#### Therapeutic potential of barriers to direct mitochondrial transplantation

**Figure 4:**
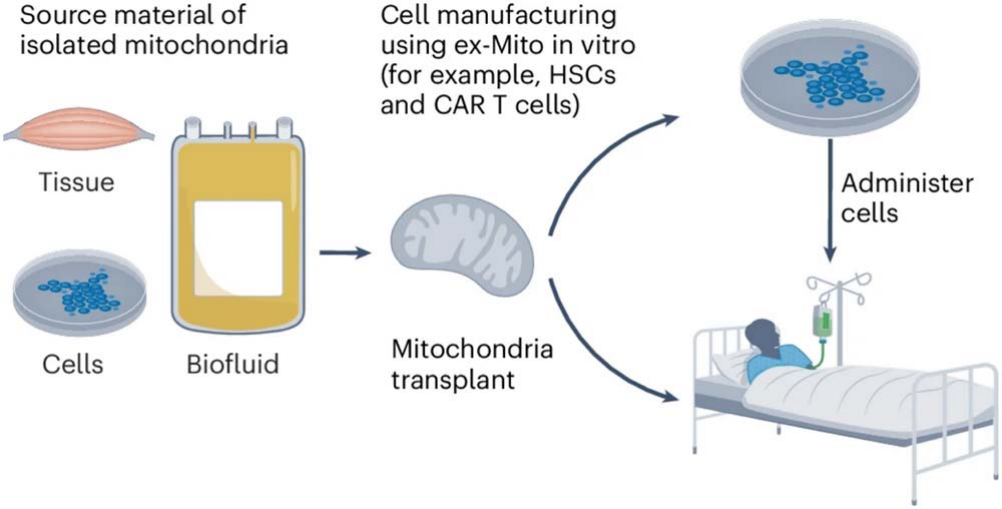
Extracellular mitochondrion-based therapies. Mitochondria isolated from cells or biofluids can be delivered directly to recipients or experimental models (mitochondrial transplantation) to restore function, a process distinct from germline-modifying mitochondrial replacement therapy. They may also be introduced into cultured cells during preparation for cell-based therapies, with excess mitochondria removed before administration. Reproduced with permission [3]. Copyright 2025, springer nature

Direct mitochondrial transplantation represents the most straightforward strategy to restore cellular bioenergetics by replenishing functional mitochondria in metabolically compromised wound tissues [3] (Figure 4). Preclinical studies have demonstrated that transplanted mitochondria can integrate into host cells, recover respiratory function, and promote tissue repair in cardiovascular and metabolic disease models [144]. To facilitate efficient delivery, multiple approaches have been developed, including direct intratissue injection, macropinocytosis-mediated uptake, and nanocarrier encapsulation, to increase mitochondrial stability and trafficking. Nevertheless, significant barriers to clinical translation persist, including potential immunogenicity, rapid degradation of exogenous mitochondria, and insufficient targeting specificity [145–147]. These limitations underscore the urgent need for innovative strategies that improve the safety, precision, and sustainability of mitochondrial transplantation, particularly within the hostile microenvironment of diabetic wounds.

#### Hydrogel-assisted mitochondrial modulation

**Figure 5:**
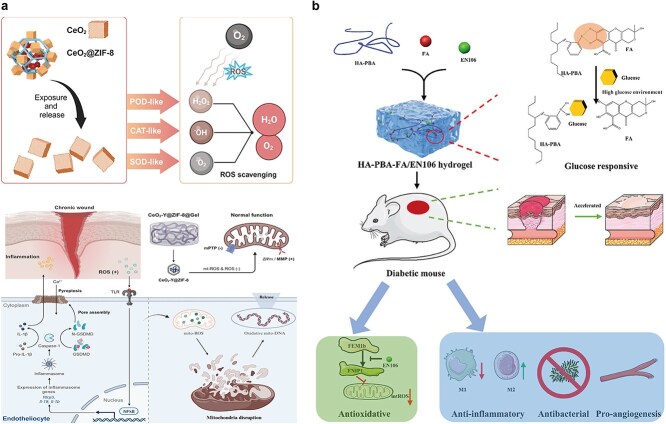
Schematic illustration of nanozyme-mediated regulation of mitochondrial redox homeostasis in diabetic wound healing. (**a**) Under diabetic conditions, excessive mitochondrial ROS (mtROS) accumulation disrupts mitochondrial integrity and impairs cellular functions in wound-relevant cells, contributing to defective angiogenesis, persistent inflammation, and delayed tissue repair. Reproduced from [37] under an open access license. (**b**) Nanozyme-based therapeutic platforms are designed to scavenge pathological mtROS and restore mitochondrial redox balance within the wound microenvironment, thereby stabilizing mitochondrial function, alleviating oxidative stress-driven cellular damage and supporting the energy-dependent processes required for coordinated wound healing. Reproduced from [148] under an open access license

Targeting pathological mitochondrial ROS and restoring redox balance constitute central therapeutic objectives in diabetic wounds, providing a mechanistic rationale for hydrogel-assisted mitochondrial modulation strategies. To overcome these challenges, bioresponsive hydrogel platforms have gained increasing attention as multifunctional systems for localized mitochondrial regulation. Smart hydrogels can be engineered to incorporate bioactive molecules such as metformin [86], Prussian blue nanoparticles (PBNPs) [143], and catalytic nanozymes [37], modulating mitochondrial dynamics, alleviating oxidative stress, and restoring redox balance. With unique properties, including self-healing ability, electrical conductivity, and responsiveness to environmental cues such as glucose and pH fluctuations, these hydrogels enable controlled and sustained therapeutic release at the wound site. In preclinical diabetic wound models, hydrogel-assisted mitochondrial modulation has been shown to suppress ROS accumulation, stimulate angiogenesis, accelerate re-epithelialization, and reestablish mitochondrial homeostasis [37, 148] (Figure 5a and b). Collectively, the integration of mitochondrial transplantation with hydrogel-based biomaterials provides a synergistic strategy with strong translational potential for improving outcomes in diabetic wound healing.

#### Nanocarriers, extracellular vesicles, and adjunctive therapies for mitochondrial modulation

In addition to hydrogels, nanotechnology-based interventions have been developed to further enhance mitochondrial targeting and function. Beyond localized antioxidant delivery, a broad range of nanocarrier- and extracellular vesicle-based strategies have been developed to restore mitochondrial function by modulating redox homeostasis, mitochondrial membrane potential, and downstream metabolic signaling pathways. Engineered nanocarriers—including acid-responsive nanoparticles [149], molybdate-oligosaccharide constructs [150], and cerium oxide-based nanoformulations [37]—have demonstrated the ability to restore mitochondrial activity, mitigate oxidative stress, and promote anti-inflammatory responses. Mechanistically, these systems increase the mitochondrial membrane potential, rebalance redox homeostasis, and activate angiogenic pathways such as the PI3K/HIF-1α/VEGF pathway [146]. In parallel, MSC-derived EVs (MSC-EVs) offer a cell-free approach for mitochondrial component transfer. MSC-EVs have been shown to regulate neutrophil polarization [151], inhibit neutrophil extracellular trap (NET) formation [19], and protect against endothelial ferroptosis, thereby enhancing OXPHOS and tissue revascularization in ischemic wounds. Moreover, small molecules such as valsartan [152] and natural compounds such as calycosin-7-glucoside can modulate macrophage metabolism and mitochondrial energetics through the AMPK, STAT6, and PI3K signaling pathways [149]. Finally, noninvasive modalities such as phototherapy—exemplified by a dual-wavelength LED patch combined with a thymoquinone/NADH hydrogel [74]—have shown potential in stimulating mitochondrial activity within macrophages and endothelial cells, ultimately promoting angiogenesis and attenuating inflammation in diabetic wound environments. In summary, these emerging strategies converge on a central theme: restoring mitochondrial homeostasis as a foundation for tissue regeneration. Continued integration of mitochondrial biology into therapeutic design holds transformative potential for the treatment of chronic diabetic wounds and broader applications in regenerative medicine.

While biomaterial-based platforms have repeatedly been associated with enhanced mitochondrial function and improved wound repair in preclinical settings, heterogeneity in material responsiveness, efficiency of mitochondrial targeting or delivery, and experimental model selection introduces substantial variability, thereby hindering direct cross-study comparison and precluding firm conclusions regarding optimal design strategies.

### The application of mitochondrion-targeted therapies in diabetic wound healing

Although primarily investigated in oncology, mitochondrion-targeted nanomedicine has demonstrated the feasibility of precisely modulating mitochondrial metabolic pathways in disease contexts, providing a conceptual and technological foundation that may be extended to wound repair-related disorders [153]. Mitochondrion-targeted therapies have emerged as promising strategies for promoting diabetic wound healing by restoring mitochondrial integrity, mitigating oxidative stress, and enhancing cellular bioenergetics. Guided by the mechanisms outlined above, mitochondrion-targeted strategies for diabetic wound healing seek to restore mitochondrial homeostasis by mitigating oxidative stress, rebalancing mitochondrial dynamics, and sustaining cellular bioenergetics.

#### Antioxidant nanozyme hydrogels for diabetic wound healing

**Figure 6:**
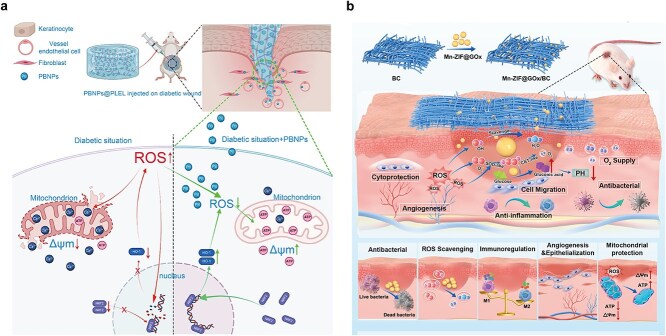
Schematic illustration of nanozyme–hydrogel-based strategies for the coordinated regulation of mitochondrial homeostasis and the wound microenvironment in diabetic wound healing. (**a**) In the diabetic wound milieu, excessive oxidative stress and persistent inflammation disrupt mitochondrial function in wound-relevant cells, including endothelial cells, fibroblasts, and immune cells, thereby impairing angiogenesis, ECM remodeling, and inflammation resolution. Reproduced with permission [143]. Copyright 2022, American Chemical Society. (**b**) Nanozyme-integrated hydrogel platforms are designed to create a permissive local microenvironment by scavenging pathological ROS and stabilizing mitochondrial function while providing sustained and localized therapeutic support. through the preservation of mitochondrial homeostasis and the modulation of inflammatory and angiogenic responses, these systems promote coordinated tissue repair. Reproduced from [154] under an open access license. *ROS* reactive oxygen species, *ECM* extracellular matrix

Antioxidant nanoformulations represent a promising therapeutic avenue for diabetic wound healing, primarily by mitigating oxidative stress and restoring mitochondrial function. Excessive mtROS is a major driver of chronic inflammation and impaired tissue repair in diabetic wounds, making redox rebalancing a primary therapeutic target [143] (Figure 6a). Xu *et al.* [143] developed a thermosensitive hydrogel incorporating PBNPs, which exhibit catalase-, peroxidase-, and superoxide dismutase-mimetic activities. These nanozymes effectively scavenge ROS, protect mitochondrial integrity, and activate NRF2/HO-1 signaling, thereby promoting angiogenesis and tissue regeneration. In a parallel strategy, a dynamic, multistage nanozyme hydrogel incorporating Mn-based nanozymes within a bacterial cellulose matrix has been reported to reestablish redox homeostasis, preserve mitochondrial function, and support cellular bioenergetic metabolism while concurrently biasing macrophage responses toward a proangiogenic immune phenotype. Such integrated regulation of oxidative stress, mitochondrial homeostasis, and immune signaling is consistent with the therapeutic framework illustrated in Figure 6b, highlighting the potential of nanozyme–hydrogel platforms to facilitate angiogenesis and tissue repair via microenvironmental reprogramming [154]. Collectively, these hydrogel-based nanoformulations act through both redox homeostasis and immune modulation, offering a dual-action platform for accelerating diabetic wound repair.

#### Self-healing hydrogels loaded with two drugs

**Figure 7:**
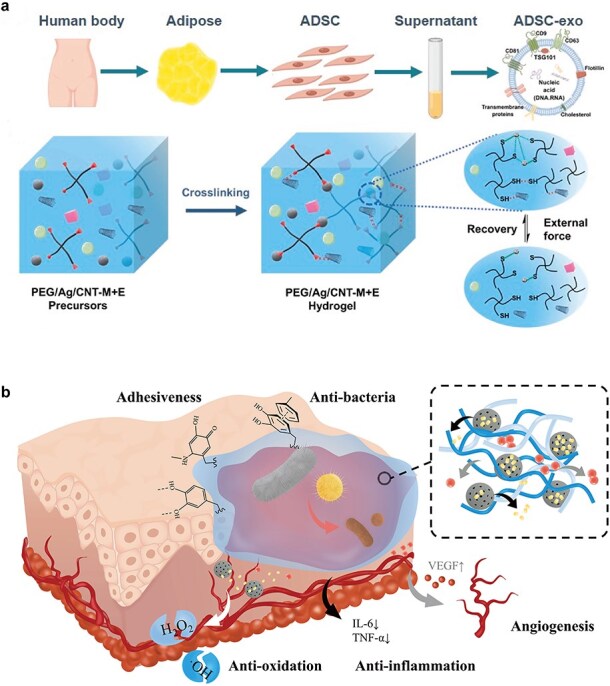
Advanced drug delivery platforms for mitochondrial protection and redox modulation in diabetic wound healing. (**a**) Schematic illustration of the PEG/Ag/CNT-M + E hydrogel preparation from ADSCs and ADSC-exos and its application in diabetic mice to enhance wound healing by promoting cell proliferation and angiogenesis while reducing ROS levels via the modulation of mitochondrial fission. Reproduced from [86] under an open access license. (**b**) Dual-drug encapsulated OCM@P hydrogels accelerate diabetic wound closure by concurrently providing antioxidative, anti-inflammatory, angiogenic, and antimicrobial benefits. Reproduced with permission [155]. Copyright 2023, Elsevier. *ADSCs* adipose-derived stem cells, *ROS* reactive oxygen species

Drug-loaded delivery systems have demonstrated significant promise in addressing the multifactorial pathogenesis of diabetic wounds. Given the multifactorial nature of mitochondrial dysfunction in diabetic wounds, combination strategies that simultaneously modulate mitochondrial dynamics, oxidative stress, and inflammatory signaling have attracted increasing attention. For example, a self-healing conductive hydrogel was designed to codeliver exosomes and metformin [86], effectively reducing mitochondrial fission and ROS accumulation, thereby preserving F-actin integrity and restoring microvascular function (Figure 7a). This dual-therapy strategy synergistically promoted angiogenesis and cell proliferation while attenuating inflammation in a hyperglycemic environment. In another study, Tan *et al.* [155] reported that a dual-drug–loaded polysaccharide-based self-healing hydrogel (OCM@P) that incorporated metformin for rapid release and curcumin for sustained delivery effectively enhanced re-epithelialization, granulation tissue formation, collagen remodeling, angiogenesis, and wound contraction in diabetic wounds through its combined antioxidative, antibacterial, and pro-regenerative properties, demonstrating significant potential as a multifunctional scaffold for regenerative medicine (Figure 7b). Collectively, these advanced platforms offer a multifaceted therapeutic approach that integrates mitochondrial protection, cytoskeletal stability, and immunomodulation, representing a compelling strategy for accelerating diabetic wound repair.

#### Biological agents and mitochondrion-targeted platforms

**Figure 8:**
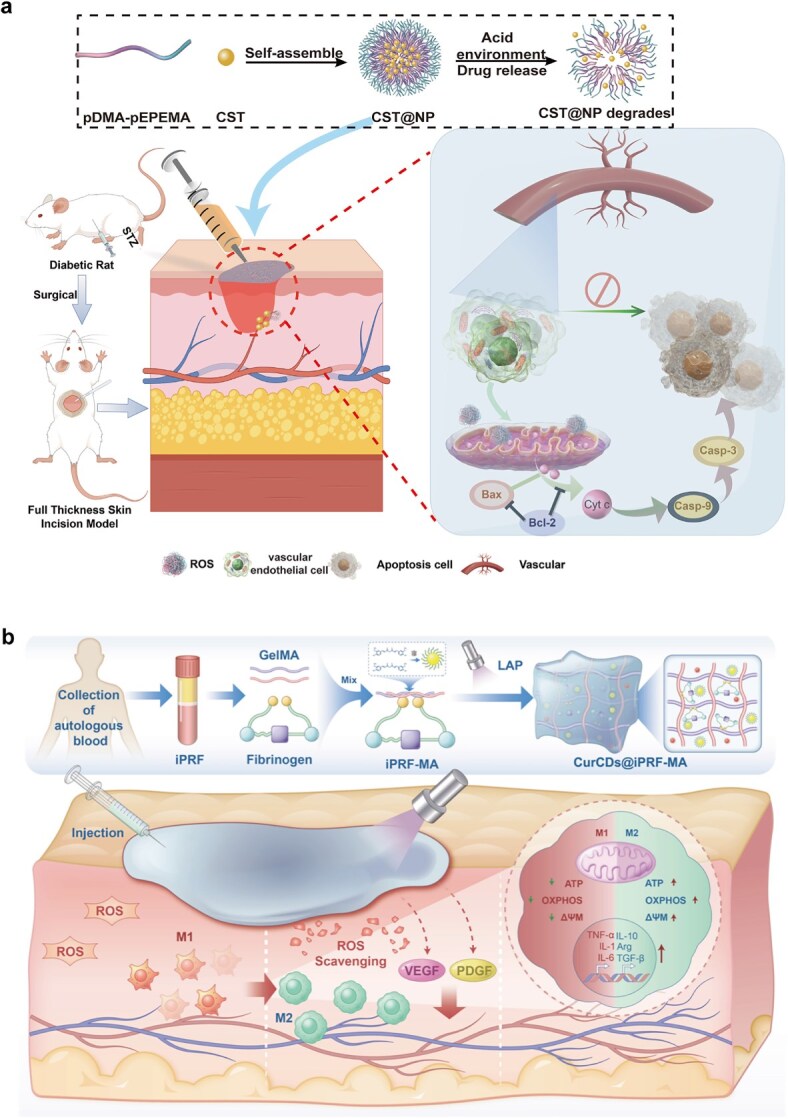
Schematic summarizing mitochondrion-targeted and biologically derived therapeutic platforms that enable mitochondrial rescue in diabetic wound healing. (**a**) Mitochondrion-targeted delivery systems, including responsive nanoparticles and engineered biomaterials, preferentially accumulate in metabolically stressed wound tissues and modulate mitochondrial homeostasis by alleviating oxidative stress, stabilizing mitochondrial function and supporting cellular bioenergetic recovery. Reproduced from [5] under an open access license. (**b**) Biologically derived platforms, such as EVs or bioactive matrices, facilitate intercellular communication by delivering mitochondrion-associated cargos or signaling cues to recipient cells, thereby promoting mitochondrial functional restoration, immunometabolic reprogramming and coordinated tissue repair. Reproduced with permission [156]. Copyright 2025, American Chemical Society. *EVs* extracellular vesicles

Biological agents and mitochondrion-targeted strategies have shown considerable promise in diabetic wound therapy because they modulate oxidative stress and promote angiogenesis. In addition to synthetic biomaterials, biologically derived agents and mitochondrion-targeted platforms have been developed to restore mitochondrial homeostasis and reprogram the inflammatory wound microenvironment. An acid-responsive nanoparticle system (CST@NPs) incorporating cortistatin within pDMA-pEPEMA carriers (Figure 8a) has been developed to address cortistatin deficiency and mitochondrial dysfunction in diabetic ulcers. Upon exposure to the acidic wound microenvironment, CST@NPs rapidly release CST, mitigating oxidative stress, reducing endothelial apoptosis, and restoring mitochondrial function [5]. This targeted delivery promotes re-epithelialization, collagen deposition, angiogenesis, and inflammation resolution, thereby markedly enhancing chronic diabetic wound repair. Similarly, an injectable, multifunctional hydrogel (CurCDs@iPRF-MA) integrating autologous platelet-rich fibrin, gelatin methacryloyl, and curcumin-derived carbogenic nanodrugs has been developed to address the limitations of conventional autologous platelet concentrates in diabetic wound repair [156]. By modulating mitochondrial homeostasis under inflammatory conditions, activating OXPHOS, and reprogramming cellular metabolism, this platform regulates macrophage polarization, scavenges excessive amounts of ROS, and enhances vascularization via the sustained release of autologous growth factors, thereby markedly accelerating chronic wound healing in patients with diabetes (Figure 8b). Together, these platforms target mitochondrial homeostasis, cytoprotection, and immune modulation to reverse microenvironmental dysfunction in diabetic wounds, offering compelling therapeutic options for chronic wound repair.

### Current challenges and limitations in mitochondrion-targeted therapies

A major challenge in translating mitochondrion-targeted therapies lies not in proof-of-concept efficacy, which is broadly supported, but in unresolved questions regarding standardization, quantitative benchmarking, and long-term functional integration of the transferred mitochondria across experimental and clinical contexts.

#### Mitochondrial dysfunction impairs diabetic wound healing

Diabetic wounds present a significant clinical challenge, primarily because of impaired angiogenesis, chronic inflammation, and oxidative stress, all of which are intricately linked to mitochondrial dysfunction [29, 107, 157]. Mitochondria play essential roles in cellular energy metabolism, redox homeostasis, and signal transduction, which are disrupted in diabetic wounds [107]. Mitochondrial dysfunction leads to excessive ROS accumulation, contributing to tissue damage and hindering proper wound healing. Despite advances in mitochondrial-targeted therapies, the exact mechanisms by which mitochondrial dysfunction exacerbates diabetic wound pathogenesis remain incompletely understood. Current treatments often fail to provide sustainable mitochondrial restoration, particularly in complex wound environments. To address these challenges, multifunctional hydrogels incorporating two therapeutic agents, such as exosomes and metformin, have emerged as promising therapeutic platforms. These dual-loaded hydrogels not only reduce mitochondrial fragmentation and ROS levels but also promote wound healing by improving microvascular function and angiogenesis [86]. Moreover, the use of glucose-responsive hydrogels for controlled drug delivery can enhance the localized and sustained therapeutic effect, offering a more efficient approach to diabetic wound management [44, 86].

#### Limitations of antioxidant-based therapies

Excessive ROS production is a key contributor to the pathophysiology of diabetic wounds. Antioxidative-based therapies, such as PBNPs incorporated into hydrogels, have shown potential for mitigating ROS levels and protecting mitochondrial function [143]. However, these therapies face limitations in achieving prolonged control over ROS homeostasis, particularly in vivo, where achieving adequate tissue penetration and sustained effects remain a challenge. Despite promising in vitro results, the clinical application of these therapies is constrained by suboptimal drug release profiles and insufficient targeting precision. Acid-responsive nanoparticles, such as CST-loaded nanoparticles (CST@NPs), offer a more localized and precise therapeutic approach. These nanoparticles respond to the acidic microenvironment of diabetic wounds, enabling rapid and targeted release of therapeutic agents. CST@NPs have been demonstrated to reduce oxidative stress, enhance mitochondrial function, and accelerate wound healing [5]. This approach, by ensuring more effective ROS scavenging and mitochondrial protection, offers a promising solution for improving the long-term treatment of chronic diabetic wounds.

#### Immunomodulation and mitochondrial repair in diabetic wound healing

The chronic inflammation observed in diabetic wounds is a critical factor contributing to delayed healing. Mitochondrial dysfunction in immune cells, such as neutrophils and macrophages, exacerbates inflammation by increasing ROS production and hindering the resolution of inflammation [151]. Moreover, mitochondrial dysfunction impairs the polarization of macrophages from the proinflammatory M1 phenotype to the tissue-repairing M2 phenotype, which is essential for angiogenesis and tissue regeneration [149]. This dysfunction impedes proper wound healing, highlighting the need for therapies that restore both mitochondrial function and immune regulation. Nanomaterials such as molybdate-oligosaccharide nanoparticles (CMO) represent a promising strategy for modulating macrophage mitochondrial function. These nanoparticles promote M2 polarization and reduce ROS levels, which in turn enhances angiogenesis and tissue repair through the PI3K/HIF-1α/VEGF pathway [146]. This approach, by simultaneously addressing mitochondrial health and immune cell polarization, has the potential to accelerate wound healing and improve the inflammatory microenvironment in diabetic wounds.

#### Challenges in mitochondrial transplantation and transfer

Mitochondrial transplantation is an emerging strategy aimed at restoring mitochondrial function through the introduction of healthy mitochondria into damaged cells. This method holds great promise for treating diseases associated with mitochondrial dysfunction, including diabetic complications. However, the clinical application of mitochondrial transplantation is hindered by several challenges, such as low transfer efficiency, poor mitochondrial stability, and difficulties in ensuring the successful integration of transplanted mitochondria into recipient cells [146, 158, 159]. These limitations present significant obstacles to the widespread use of this technique for wound healing. Advances in mitochondrial delivery technologies, such as EVs or liposomes, may improve mitochondrial transfer efficiency and stability. These systems increase mitochondrial targeting and biocompatibility, facilitating more effective mitochondrial restoration in damaged cells. By combining mitochondrial transplantation with advanced drug delivery platforms, such approaches could address the challenges of mitochondrial stability and cellular integration, thereby enhancing the therapeutic potential of mitochondria-based treatments for diabetic wound healing [147].

#### Translational barriers and future directions

Despite growing interest in mitochondrion-targeted strategies for diabetic wound healing, clinical translation remains constrained by inefficient delivery, limited durability, and challenges in achieving sustained mitochondrial restoration within the hostile wound microenvironment. Current approaches, including hydrogels, nanoparticles, and mitochondrial transfer or transplantation, show promise but often lack scalability, targeting precision, and long-term functional integration [5, 61, 156]. Importantly, a recent consensus framework has highlighted the urgent need for standardized terminology and classification of mitochondrial transfer and transplantation, providing a conceptual foundation for improving mechanistic clarity and cross-study comparability [3]. Within this context, the absence of harmonized definitions, quantitative benchmarking of transfer efficacy, and systematic safety evaluation remains a major translational barrier. Addressing these gaps will require rigorously designed, context-specific experimental frameworks that integrate mitochondrial biology with wound pathophysiology. Collectively, these efforts are essential for advancing mitochondrion-targeted interventions toward clinically meaningful applications in diabetic wound repair.

## Conclusions

Mitochondrial dysfunction plays a central pathophysiological role in diabetic wound chronicity, driving a self-reinforcing cascade of chronic inflammation, oxidative stress, and endothelial dysfunction that collectively impedes effective tissue repair. Emerging therapeutic strategies targeting mitochondrial rescue—encompassing antioxidant-loaded nanoparticle systems, multifunctional hydrogels, and EV-based platforms—have demonstrated compelling preclinical efficacy in restoring redox homeostasis, promoting angiogenesis, and recalibrating immune responses within the diabetic wound microenvironment. Of particular translational significance, intercellular mitochondrial transfer mediated via TNTs, EVs, GJs and cell fusion, together with direct mitochondrial transplantation facilitated by bioresponsive delivery systems, has been identified as a mechanistically distinct and therapeutically promising avenue for restoring bioenergetic capacity in metabolically compromised recipient cells. Concurrently, noninvasive adjunctive modalities—including wearable phototherapy devices and stimuli-responsive drug delivery platforms—offer clinically attractive complements to biomaterial-based interventions by enabling spatiotemporally controlled mitochondrial support at the wound site. Nevertheless, critical translational barriers persist, including suboptimal mitochondrial transfer efficiency, unresolved immunogenicity concerns, and insufficient evidence regarding the long-term functional integration and stability of transplanted mitochondria in the hyperglycemic milieu. Addressing these challenges through rigorously designed preclinical models, standardized characterization frameworks, and well-powered clinical investigations will be essential to translate mitochondrion-centered therapeutic strategies from experimental platforms into evidence-based clinical practice, ultimately advancing the standard of care for patients with diabetic wounds and patients with other types of chronic wound conditions.

## Supplementary Material

AFigure_legend_tkag018
