# Supplementary material for: Mitochondrion-targeted therapies for diabetic wound healing: from mechanism to therapeutic opportunity
Source: Burns Trauma. 2026 Mar 2;14:tkag018. doi: 10.1093/burnst/tkag018 (PMC13245933; doi:10.1093/burnst/tkag018)
Supplement: AFigure_legend_tkag018 [file afigure_legend_tkag018.docx]

Figure legend

**Figure 1. Schematic illustration of mitochondrial dysfunction in diabetic wound healing.** A magnified mitochondrion within a semitransparent cell highlights complexes I–IV, with complexes I and III impaired. Hyperglycemia negatively affects these complexes, lowering ATP levels and impairing fibroblasts, keratinocytes, and endothelial cells. Electron leakage from complexes I and III generates ROS, causing lipid, protein, and mtDNA damage while triggering inflammation and senescence. Mitochondrial quality control is disrupted by the suppression of PGC-1α, increased expression of Drp1, decreased expression of MFN2, and reduced expression of PINK1/Parkin, leading to the accumulation of damaged mitochondria. This integrated diagram visually summarizes the multifactorial mitochondrial impairments contributing to delayed diabetic wound healing. ATP: adenosine triphosphate, ROS: reactive oxygen species, mtDNA: mt deoxyribonucleic acid, PGC-1α: peroxisome proliferator-activated receptor gamma coactivator 1-alpha, Drp1: dynamin-related protein 1, MFN2: mitofusin 2, PINK1: PTEN-induced kinase 1. Created with Adobe Illustrator

**Figure 2.** **Overview of mitochondrial quality control.** Mitochondrial function is preserved through an integrated network of regulatory processes, including biogenesis, fusion, fission, and mitophagy. Biogenesis, largely driven by PGC-1α, promotes the generation of new mitochondria to meet cellular energy demands. Fusion, mediated by MFN1, MFN2, and OPA1, facilitates the exchange of mitochondrial contents, supporting functional complementation and adaptation to stress. Fission, coordinated by Drp1 and its adaptors (FIS1, MFF, MID49, and MID51), enables mitochondrial distribution, segregation of damaged organelles, and cellular remodeling. Mitophagy, which is governed primarily by the PINK1–Parkin pathway, selectively removes dysfunctional mitochondria through lysosomal degradation. Together, these processes maintain mitochondrial integrity, bioenergetic capacity, and cellular homeostasis, which are critically involved in tissue repair and pathological conditions. PGC-1α: peroxisome proliferator-activated receptor gamma coactivator 1-alpha, MFN: mitofusin, OPA1: optic atrophy 1, FIS1: fission, mitochondrial 1, MFF: mitochondrial fission factor, MID: mitochondrial dynamics protein, PINK1: PTEN-induced kinase 1. Created with Adobe Illustrator

**Figure** **3. Modes of intercellular mitochondrial transfer.** a. Tunneling nanotubes (TNTs) establish direct cytoplasmic continuity between donor and recipient cells via actin filaments and microtubules, enabling active mitochondrial trafficking driven by molecular motors including myosin, kinesin, and dynein, in coordination with adaptor proteins of the Miro/TRAK (Trak1/Trak2) complex. b. Extracellular vesicles (EVs) mediate long-range mitochondrial exchange between spatially separated cells. Mitochondria encapsulating EVs are generated by budding or secretion from donor cells and subsequently internalized by recipient cells through plasma membrane fusion, enabling mitochondrial delivery across substantial intercellular distances without direct cell contact. c. Gap junctions (GJs) facilitate organelle transfer at sites of direct cell–cell contact. Cx43 assembled GJs at cell–cell contact sites form intercellular channels that permit the passage of mitochondria or mitochondria-derived components between adjacent cells. d. Cell fusion represents an additional mechanism for mitochondrial sharing. Mitochondrial sharing occurs during transient or permanent plasma membrane fusion between two cells, a process dependent on the surface expression of fusion protein, most notably syncytins which are required to drive complete membrane merger and cytoplasmic exchange. Miro: mitochondrial Rho, Trak: trafficking kinesin protein, Cx43: connexin 43. Created with Adobe Illustrator

**Figure 4. Extracellular mitochondrion-based therapies.** Mitochondria isolated from cells or biofluids can be delivered directly to recipients or experimental models (mitochondrial transplantation) to restore function, a process distinct from germline-modifying mitochondrial replacement therapy. They may also be introduced into cultured cells during preparation for cell-based therapies, with excess mitochondria removed before administration. Reproduced with permission[3]. Copyright 2025, Springer Nature. Created with Adobe Illustrator

**Figure 5. Schematic illustration of nanozyme-mediated regulation of mitochondrial redox homeostasis in diabetic wound healing.** A. Under diabetic conditions, excessive mitochondrial reactive oxygen species (mtROS) accumulation disrupts mitochondrial integrity and impairs cellular functions in wound-relevant cells, contributing to defective angiogenesis, persistent inflammation, and delayed tissue repair. Reproduced from[37] under an open access license. B. Nanozyme-based therapeutic platforms are designed to scavenge pathological mtROS and restore mitochondrial redox balance within the wound microenvironment, thereby stabilizing mitochondrial function, alleviating oxidative stress-driven cellular damage and supporting the energy-dependent processes required for coordinated wound healing. Reproduced from[148] under an open access license. Created with Adobe Illustrator

**Figure 6. Schematic illustration of nanozyme–hydrogel-based strategies for the coordinated regulation of mitochondrial homeostasis and the wound microenvironment in diabetic wound healing.** A. In the diabetic wound milieu, excessive oxidative stress and persistent inflammation disrupt mitochondrial function in wound-relevant cells, including endothelial cells, fibroblasts, and immune cells, thereby impairing angiogenesis, extracellular matrix remodeling, and inflammation resolution. Reproduced with permission[143]. Copyright 2022, American Chemical Society. B. Nanozyme-integrated hydrogel platforms are designed to create a permissive local microenvironment by scavenging pathological reactive oxygen species and stabilizing mitochondrial function while providing sustained and localized therapeutic support. Through the preservation of mitochondrial homeostasis and the modulation of inflammatory and angiogenic responses, these systems promote coordinated tissue repair. Reproduced from[154] under an open access license. Created with Adobe Illustrator

**Figure 7. Advanced Drug Delivery Platforms for Mitochondrial Protection and Redox Modulation in Diabetic Wound Healing.** A. Schematic illustration of the PEG/Ag/CNT-M + E hydrogel preparation from ADSCs and ADSC-Exos and its application in diabetic mice to enhance wound healing by promoting cell proliferation and angiogenesis while reducing ROS levels via the modulation of mitochondrial fission. Reproduced from[86] under an open access license. B. Dual-drug encapsulated OCM@P hydrogels accelerate diabetic wound closure by concurrently providing antioxidative, anti-inflammatory, angiogenic, and antimicrobial benefits. Reproduced with permission[155]. Copyright 2023, Elsevier. ADSCs: adipose-derived stem cells, ROS: reactive oxygen species. Created with Adobe Illustrator

**Figure 8.** **Schematic summarizing mitochondrion-targeted and biologically derived therapeutic platforms that enable mitochondrial rescue in diabetic wound healing.** A. Mitochondrion-targeted delivery systems, including responsive nanoparticles and engineered biomaterials, preferentially accumulate in metabolically stressed wound tissues and modulate mitochondrial homeostasis by alleviating oxidative stress, stabilizing mitochondrial function and supporting cellular bioenergetic recovery. Reproduced from[5] under an open access license. B. Biologically derived platforms, such as extracellular vesicles or bioactive matrices, facilitate intercellular communication by delivering mitochondrion-associated cargos or signaling cues to recipient cells, thereby promoting mitochondrial functional restoration, immunometabolic reprogramming and coordinated tissue repair. Reproduced with permission[156]. Copyright 2025, American Chemical Society. Created with Adobe Illustrator
